# Is There a Link between Mitochondrial Reserve Respiratory Capacity and Aging?

**DOI:** 10.1155/2012/192503

**Published:** 2012-06-05

**Authors:** Claus Desler, Thomas Lau Hansen, Jane Bruun Frederiksen, Maiken Lise Marcker, Keshav K. Singh, Lene Juel Rasmussen

**Affiliations:** ^1^Center for Healthy Aging, Department of Cellular and Molecular Medicine, University of Copenhagen, 2200 Copenhagen, Denmark; ^2^Department of Genetics, School of Medicine, University of Alabama at Birmingham, Birmingham, AL 35294, USA

## Abstract

Oxidative phosphorylation is an indispensable resource of ATP in tissues with high requirement of energy. If the ATP demand is not met, studies suggest that this will lead to senescence and cell death in the affected tissue. The term reserve respiratory capacity or spare respiratory capacity is used to describe the amount of extra ATP that can be produced by oxidative phosphorylation in case of a sudden increase in energy demand. Depletion of the reserve respiratory capacity has been related to a range of pathologies affecting high energy requiring tissues. During aging of an organism, and as a result of mitochondrial dysfunctions, the efficiency of oxidative phosphorylation declines. Based on examples from the energy requiring tissues such as brain, heart, and skeletal muscle, we propose that the age-related decline of oxidative phosphorylation decreases the reserve respiratory capacity of the affected tissue, sensitizes the cells to surges in ATP demand, and increases the risk of resulting pathologies.

## 1. Introduction

In aerobic cells the majority of ATP is produced by oxidative phosphorylation. This process takes place in the mitochondria where electrons that are donated from the Krebs cycle are passed through the four complexes (complex I–IV) comprising the electron transport chain (ETC), eventually reducing oxygen and producing water. The flux of electrons creates an electrochemical potential between the inter-membrane space and the matrix of the mitochondria. This potential is utilized by the ATP synthase to phosphorylate ADP producing ATP.

Many cells operate at a basal level that only requires a part of their total bioenergetic capability. The difference between ATP produced by oxidative phosphorylation at basal and that at maximal activity is termed “spare respiratory capacity” or “reserve respiratory capacity”, where the latter denomination will be used throughout this review. Under certain conditions a tissue can require a sudden burst of additional cellular energy in response to stress or increased workload. If the reserve respiratory capacity of the cells is not sufficient to provide the required ATP affected cells risk being driven into senescence or cell death. Exhaustion of the reserve respiratory capacity has been correlated with a variety of pathologies including heart diseases [1], neurodegenerative disorders [2, 3], and cell death in smooth muscle [4].

According to the mitochondrial theory of aging, functional alterations in mitochondria contribute to the aging process [5]. The mitochondrion is the only organelle in animal cells that has a distinct genome and even though mitochondria are involved in metabolism of fatty acids, amino acids, and lipids, initiation of apoptosis, and other essential cellular functions, the human mitochondrial genome exclusively encodes 24 peptides needed for mitochondrial protein synthesis and 13 essential subunits of the ETC and ATP synthase. The encoded polypeptides comprise few but essential subunits of complex I (7 peptides), complex III (1 peptide), complex IV (3 peptides), and ATP synthase (2 peptides) [6, 7]. It has been demonstrated that brain, heart, and skeletal muscle of aging humans harbor an increased mutational load of the mitochondrial genome when compared to corresponding tissues of young [8–10]. Mutations in mtDNA affect the function of the ETC, the electrochemical potential, and the generation of ATP by oxidative phosphorylation. Furthermore, mutations of mtDNA can result in an elevated production of reactive oxygen species (ROS). ROS serve as second messenger molecules but an overproduction can, as we have reviewed earlier [11], lead to unwanted oxidation of lipids, proteins, and DNA which can further lead to cellular damage, mutations, and cell death. ROS generated by mitochondria have, therefore, numerous times been appointed as the mediator of the mitochondrial contribution to the aging process [12–14]. Even though this relationship in some contexts has been validated, studies have demonstrated that mitochondrial generated ROS cannot be the sole explanation for the correlation between aging and mitochondria. A strong relation between the fitness of the mtDNA, premature aging, and age-related pathologies has been demonstrated with the independent construction of two mouse lines expressing mutated versions of the mitochondrial polymerase gamma [15, 16]. Both mouse lines developed an mtDNA mutator phenotype, which was associated with a decreased oxidative phosphorylation but neither displayed an increase in ROS production. Nevertheless, the two mouse lines displayed symptoms of premature aging and reduced lifespan in the form of weight loss, alopecia, osteoporosis, kyphosis, cardiomyopathy, anemia, gonadal atrophy, and sarcopenia. The median lifespan was reduced to 48 weeks and maximum lifespan of 61 weeks when compared to the typical wildtype lifespan of 2 years [15, 16]. In human fibroblasts and human endothelial cells it has been demonstrated that chronic exposure to agents uncoupling the mitochondrial membrane potential forces the cells into premature senescence [17, 18]. Treating the cells with an antioxidant eliminated increased ROS production but did not prevent premature senescence [17, 19]. Rather the premature senescence was found to be the result of a decrease in the level of oxidative phosphorylation [19].

In this paper we hypothesize that mitochondria contributes to aging and age-related pathologies through a life-long continued decrease of the respiratory reserve capacity. The decrease sensitizes high energy requiring tissues to an exhaustion of the reserve respiratory capacity. This increases the risk of a range of pathologies that correspondingly are known to be age-related. Through a review of the effects of aging on the regulation of oxidative phosphorylation, we wish to substantiate this hypothesis. In addition, by using brain, heart, and skeletal muscle as examples, we will review how an age-related decrease of the reserve respiratory capacity is implicated in a variety of pathologies in the affected tissues.

## 2. Regulation of Reserve Respiratory Capacity

The energy requirement of different tissues and cells fluctuates constantly and the metabolism of ATP is correspondingly regulated to avoid futile energy expenditure and to cater the specific needs of different tissues. In accordance, it has for almost 60 years been known that the rate of mitochondrial respiration increases when mitochondria synthesize more ATP implying a feedback mechanism linking cellular ATP demand to regulation of oxidative phosphorylation [20]. Today this feedback mechanism is better described, and it is demonstrated to contain regulatory components that can be divided into short- and long-term regulators.

Short-term regulators of the oxidative phosphorylation exert their control in response to sudden changes of ATP demand. Cytochrome *c* oxidase (Complex IV of the ETC) has been demonstrated to be a potent facilitator of short-term regulation [21]. Complex IV is the final electron acceptor of the ETC and catalyzes the reduction of O_2_ to H_2_O.The complex has been demonstrated to be allosterically inhibited by ATP whereby a futile overproduction of ATP is avoided [22, 23]. Furthermore, the catalytic activity of complex IV is regulated by the mitochondrial electric membrane potential [24, 25], balancing the activity of the ETC according to the needs, while avoiding a hyperpolarization of the mitochondrial membrane and, thereby, avoiding an excessive ROS production [23]. Finally, nitric oxide (NO) has at submicromolar concentrations been demonstrated to act as a competitive inhibitor of complex IV and inhibit electron transport at complex III [26–28]. Mitochondrial NO is produced via the mitochondrial nitric oxide synthase (mtNOS), which is located in the inner mitochondrial membrane [29]. MtNOS physically interacts with both complexes I and IV [30, 31]. It is generally believed that mtNOS is a voltage-dependent enzyme whose generation of NO is regulated by mitochondrial membrane potential according to the energy requirement of the cell which in turn regulates the activity of the ETC [32, 33]. Several other targets exist for posttranslational regulation of the ETC and the ATP synthase. Multiple phosphorylation sites have been demonstrated in complex IV [34–36] and phosphorylation of these sites has been shown to almost completely inhibit the catalytic activity of the complex [35]. Similarly, complex I and the ATP synthase have been demonstrated to contain phosphorylation sites [37, 38]. The mtNOS is similarly post-transcriptionally regulated by both acylation and phosphorylation [39].

Long-term regulators of the oxidative phosphorylation are effectors that alter the mitochondrial respiration in response to a changing role for the mitochondria in the tissue, for example, in skeletal muscles after endurance training [40] or as demonstrated as a consequence of caloric restriction [41]. Interestingly, caloric restriction has in the cerebella of mice been correlated with an increased reserve respiratory capacity [42]. This increase is proposed to be the result of an enhanced expression of the nuclear nitric oxide synthase (nNOS) and increased levels of nuclear NO which, in contrast to mitochondrial produced NO, is proposed to increase respiratory capacity by affecting the transcriptional coactivator PGC-1*α* [42]. Together with PGC-1*β*, PGC-1*α* is known to be involved in regulation of mitochondrial biogenesis [43].

Long-term regulators can also permanently change the properties of the mitochondrial respiration. This allows a differentiation of mitochondria making them specialized for different cells and tissues. Accordingly, the ratio between produced ATP and consumed oxygen can vary greatly between different tissues. Heart and brain mitochondria of rats have been demonstrated to produce ATP faster than mitochondria of the liver while liver mitochondria produce ATP more efficiently using less oxygen per produced ATP [44]. This is believed to be due to the expression of different isoforms of specific subunits of the ETC. Such isoforms are known for cytochrome *c* of the ETC and complex IV. Accordingly it has been found that cytochrome *c* is expressed as a somatic and a testes-specific isoform in most mammals [45]. Complex IV isolated from cow lung additionally showed a 2.5-fold increased activity compared to liver complex IV [46]. It has further been demonstrated that different isoforms of complex IV can explain differences in kinetic behavior of the enzyme in mouse cortical astrocytes and cerebellar granule cells [47].

By definition, the reserve respiratory capacity is the difference between ATP produced by oxidative phosphorylation at basal and at maximal respiratory activity. Regulation of the oxidative phosphorylation is divided into short- and long-term regulators. Short-term regulators mediate the oxidative phosphorylation according to the immediate energy requirement. Short-term regulators are, therefore, important for the regulation of the basal activity of ATP production. Long-term regulators of the oxidative phosphorylation modulate the properties of the oxidative phosphorylation and are correspondingly essential for the setting of the maximal respiratory capacity. In extension of this, the reserve respiratory capacity is defined by the interplay between the short- and long-term regulators of the oxidative phosphorylation.

## 3. Aging and Oxidative Phosphorylation

Aging impairs mitochondrial function by affecting both the capacity and the control of oxidative phosphorylation. Reduced activities of complexes I and IV, but not complexes II and III, have been found in aging mice and rats. In brain tissue of aging rats a 22–35% reduction of complex IV activity was measured [48]. A similar trend has been demonstrated in humans where a reduced activity of complex IV has been found in skeletal muscle, heart, and brain of aging subjects [49–52]. Correspondingly, an age-related decline of mitochondrial capacity for oxidative phosphorylation has been demonstrated in both human skeletal muscle and rat hearts [9, 52, 53]. One factor affecting oxidative phosphorylation is an age-related accumulation of mtDNA mutations resulting in an aberrant expression of mitochondrial encoded ETC subunits [9, 52]. Increasing evidence suggests an important role of accumulating mtDNA mutations in the pathogenesis of many age-related neurodegenerative diseases as well as a number of age-related pathological alterations of heart, skeletal muscle, and the vascular system [54–56]. MtDNA is more susceptible to DNA damages in comparison to nuclear DNA [57]. mtDNA molecules are localized in close proximity to the ETC, which continuously generates ROS. Thus, the mutation rate of mtDNA has been reported to be up to 15-fold higher than that observed for nuclear DNA in response to DNA damaging agents [57]. Cumulative damage to the mtDNA is however, not the only contributor to the age-related decline of oxidative phosphorylation. Transcriptional profiling has revealed different regulation of nuclear genes encoding important peptides for oxidative phosphorylation when comparing young to old. In human frontal cortex the *α*-subunit of the ATP synthase was found to be significantly downregulated in old when compared to young [58]. Using siRNA to approximate same expression levels of *α*-subunit of the ATP synthase in a neuroblastoma cell line resulted in a 24% reduction in cellular ATP levels [58]. In another study, skeletal muscle from aging rhesus monkeys displayed a selective down-regulation of nuclear-encoded proteins involved in mitochondrial electron transport and oxidative phosphorylation [59]. These included subunits of complex I, complex IV, and ATP synthase. One mechanism that has been proposed to link aging with decreased transcription of nuclear genes encoding mitochondrial peptides is the shortening of telomeres [60]. Telomerase deficient mice were intercrossed to produce successive generations with decreasing telomere reserves. The shortened telomeres of subsequent generations were demonstrated to be associated with reduced mtDNA content and impaired oxidative phosphorylation in the hematopoietic stem cells, livers, and hearts of the telomerase deficient mice [60]. The telomere dysfunction was associated with a repression of the transcriptional coactivators PGC-1*α* and PGC-1*β*, and this repression was hypothesized to be responsible for the observed decline in mitochondrial function [60].

An age-related decline of oxidative phosphorylation can therefore be related to a decline in expression of both mitochondrial and nuclear expressed peptides functioning in the ETC. This decline affects most components of the oxidative phosphorylation. Of the affected components, complex IV of the ETC stands out as it has an important role in regulation of the oxidative phosphorylation. Complex IV is the primary target of regulation by both short-term and long-term regulators, and the complex is therefore the main determinant of the maximal, basal, and thereby also the reserve respiratory capacity of mitochondria. Nuclear encoded subunits of complex IV have been shown to be downregulated in an age-related manner [59]. Of the 13 peptides constituting complex IV, three are mitochondrial encoded [6, 7]. Consequently, an age-related decline of mtDNA fidelity also impairs the activity and the regulatory properties of complex IV and subsequently impairs, or inactivates the ETC in all of the tissues reviewed in this paper.

## 4. Reserve Respiratory Capacity in Neuronal Diseases

Neurodegenerative diseases including Alzheimer's disease, Parkinson's disease and Huntington's disease, all have a major impact on personal lives as well as the society.

As reviewed by several, the mitochondrial function is recognized as a causal factor in the pathogenesis of several neuronal diseases [61–63]. The oxidative phosphorylation and, thereby, respiratory capacity is critical for neuronal susceptibility to cellular stress caused by hypoxia, nutrients depletion, or excitatory stimuli by neurotransmitters [64, 65]. The dependency of the human brain on oxygen for oxidative phosphorylation is emphasized by the fact that the human brain consumes approximately 16% of all oxygen absorbed, but only accounts for 2% of the entire body mass [66]. Furthermore, exhausting the reserve respiratory capacity of a neuron can have fatal consequences. Resting neurons utilize approximately 6% of its maximal respiratory capacity, while firing neurons utilize up to 80% [67]. Therefore, subtle decreases in reserve respiratory capacity from aging increase neuronal vulnerability towards bioenergetic exhaustion, predisposing the tissue for diseases. This is especially evident in situations where the activity of complexes I and IV is reduced [2, 68].

Rodent models of the neurodegenerative Alzheimer's disease shows that deficiency in mitochondrial respiration precedes the pathology of the disease [69]. Alzheimer's disease is also accompanied by decreased expression and activity of enzymes involved in mitochondrial bioenergetics, as well as a generalized shift towards ketone body metabolism [70]. Correspondingly, a decline of brain metabolism is detectable in Alzheimer's disease patients as early as a decade before diagnosis [66].

Substantiating the importance of reserve respiratory capacity in neuronal pathologies, evidence for a common underlying mechanism reviewed by Nicholls and Budd in 2000 [71] and Rasola and Bernardi in 2011 [72] connects the pathogenesis of ischemic reperfusion injury and neurodegenerative diseases. This mechanism is initiated by the depletion of ATP, a result of ischemic conditions or perturbated reserve respiratory capacity [64], as reviewed by Fiskum et al. in 1999 [65].

## 5. Neurons and Aging

The greatest risk factor in the course of neurodegenerative diseases is advancing age as reviewed by Wallace et al. 1995, Beal 1995, and Bishop et al. 2010 [61, 63, 73]. Mitochondrial deletions and point mutations have been demonstrated to accumulate in neuronal tissue with normal aging [74, 75]. Studies of age-related accumulation of oxidative damage to both nDNA and mtDNA showed a preference for accumulation of damages in mtDNA with increasing age, affecting components of the oxidative phosphorylation [76]. Therefore, mutations and deletions in mtDNA have been suggested to be responsible for the aging-related factor in some neurodegenerative diseases [61, 62, 77–79].

These studies support a lately revived hypothesis—that neuronal cell death, in this context, primarily is induced by an energy crisis as ATP demands exceed the maximal respiratory capacity [80].

As reviewed by Lin and Beal in 2006 [79], a common factor in sporadic and genetically predisposed neurodegenerative diseases is the gradual perturbation of the ETC resulting in decreased activity of the oxidative phosphorylation and hence, the maximal respiration. As aging has been correlated to this, it therefore sensitizes neurons towards acute and chronic stress, lowering the threshold for the amount of damage the tissue can endure.

## 6. Reserve Respiratory Capacity in Heart Disease

By using a surgical intervention of the heart known as an aortic banding, it is possible to induce a pressure overload resulting in acute and chronic stress of the cardiac tissue. In pigs, aortic banding induces a condition mimicking hypertrophy. When investigating the resulting hypertrophic hearts *in vivo,* the reserve respiratory capacity was demonstrated to be completely exhausted [81]. Similar results were obtained from cardiac muscle tissue of rats [82–84] and in cardiomyocytes treated with oxidized lipids [4]. The loss of reserve respiratory capacity led to cell death or organ dysfunction in the studied subjects.

The mechanism behind this fatal bioenergetic exhaustion is not fully elucidated. Decreased expression and activity of complex I and complex IV have been reported in hypertrophic cardiomyopathy and murine hearts with myocardial infarct, suggesting impaired ETC [82, 83, 85–87], and a possible cause to the reduced reserve respiratory capacity observed in the failing heart. Even though reductions of ETC activity have been found to cooccur with large-scale deletions in mtDNA [87, 88], the inflicted cells only constitute a fraction of the tissue. The impairment of the ETC is, therefore, generally not believed to be caused by a change in mtDNA content or quality [82, 88]. Rather it has been proposed that the mitochondrial transcription factor A (TFAM) and the corresponding nuclear respiratory factor 2 (NRF-2) are downregulated resulting in a decreased transcription of mitochondrial DNA [82, 84]. TFAM and NRF-2 are downstream targets of the transcriptional coactivator PGC-1*α*. Accordingly, polymorphisms of PGC-1*α* have been correlated with an increased risk of hypertrophic cardiomyopathy [89]. Furthermore, knockout of PGC-1*α* in mice leads to decreased expression of ETC genes in the heart and demonstrates a gene regulatory pattern comparable to mice with hypertrophy following aortic constriction [90]. Even though a downregulation of PGC-1*α* partly can explain the demonstrated impaired ETC in the diseased heart, mitochondrial dysfunction has been demonstrated in hypertrophied hearts without a corresponding downregulation of PGC-1*α* [84]. In these cases a significant downregulation of PPAR*α* was demonstrated [84]. PPAR*α* is the key transcription factor for fatty acid oxidation.

A common feature of heart diseases is an inhibition of the ETC resulting in a decreased activity of the oxidative phosphorylation and of the maximal respiration. This response is not the consequence of mtDNA deterioration, but rather a reduced transcription of mtDNA encoded genes. Where an understanding of the pathway regulating this mechanism is emerging, the benefits of such a response remains unclear. Maybe it is an attempt to avoid the production of ROS as demonstrated during ischemia [91].

No matter what the purpose, acute and chronic stress in cardiac tissue inhibits the ETC and decreases reserve respiratory capacity of the tissue. The lowered capacity makes the heart more vulnerable to a bioenergetic exhaustion and thereby increases the risk of inducing cell death and organ failure. Aging has been demonstrated to reduce the fidelity of myocardial mtDNA resulting in a reduction of maximal respiratory capacity. Aging therefore further sensitizes the heart to acute and chronic stress, lowering the threshold of damage a heart can endure.

## 7. Skeletal Muscle and Aging

The progressive loss of skeletal muscle mass and strength observed in older individuals is a condition known as sarcopenia. The condition has been correlated with aging as well as mitochondrial dysfunction [92]. The mitochondrial reserve capacity is essential for skeletal muscle, and it has been demonstrated that an exhaustion of the reserve respiratory capacity, induced by diamides, causes cell death in vascular smooth muscle of rats [4]. Similarly, proofreading deficient polymerase gamma mice has been used to model mitochondrial related sarcopenia. The mice developed an mtDNA mutator phenotype and several signs of premature aging, including a significant loss of skeletal muscle. The study demonstrated an increased spontaneous mutation rate in the mtDNA correlated with a 35–50% reduction in the content of complex I, III, and IV, a decrease of oxidative phosphorylation and, thereby, a lower ATP content and reserve respiratory capacity [93]. The decrease of oxidative phosphorylation ultimately resulted in an induction of apoptosis in the tissue and finally sarcopenia allowing the authors to conclude that mtDNA mutations can have a causal role in sarcopenia [93].

Several large-scale studies on skeletal muscle biopsies from humans of ages ranging from 17 to 91 years have shown a significant age-related decline in mitochondrial respiratory capacity [52, 94, 95]. One study demonstrated a 50% downregulation of mitochondrial oxygen capacity in elderly compared to adult skeletal muscle [94]. The reduced capacity of oxidative phosphorylation can partly be explained by an increase in mtDNA mutations and a significant down-regulation of subunits of complex I and IV demonstrated in the aging skeletal muscle of humans [9, 96–99]. It has been proposed that the aging muscle compensates for the lower mitochondrial respiratory capacity by upregulation of the mitochondrial content to avoid rate-limiting ATP production [99]. Accordingly, ragged red fibers harboring an abnormal accumulation of mitochondria have been shown to occur in aging skeletal muscle [100]. The occurrence of ragged red fibers was investigated in biopsies of skeletal muscle obtained from humans at various ages. Ragged red fibers were demonstrated to be extremely rare in subjects under the age of 40 years, while they were demonstrated in more than half of the subjects between the age of 40 and 50 years, and in almost all of the subjects older than the age of 50 years [100]. The biopsies were stained for complex IV activity and it was demonstrated that in subjects under the age of 40 years, none, except an outlier, presented complex IV negative fibers. In subjects between the age of 40 and 50 years only 21% carried complex IV negative fibers, and almost all subjects above the age of 50 years displayed complex IV negative fibers [100]. This indicates that even though the cells attempt to compensate for a low mitochondrial respiratory capacity by upregulating mitochondrial content, this is only possible until the age of 50 as demonstrated by the occurrence of complex IV negative fibers.

The complex IV negative fibers were demonstrated to be associated with a decrease of mtDNA content [100]. This was also shown in a separate study where mtDNA content of human skeletal muscle was demonstrated to decrease in an age-related fashion. This study also determined an age-related decrease of both nuclear and mitochondrial expression of genes encoding subunits of complex IV and this decrease was correlated with a corresponding decrease of mitochondrial ATP production rate. Together this led the authors to suggest that the declining mitochondrial function could contribute to the low physical function that is common in older people [9].

## 8. Skeletal Muscle and Physical Activity

Increasing evidence shows that the age-related loss of muscle strength can be avoided, or the muscle strength can be regained by regular endurance exercise [97, 101]. Several studies have investigated the effect of exercise on the mitochondrial metabolism in aging. It has been demonstrated that the mitochondrial ATP production rate in skeletal muscle was significantly lowered with age in groups having a sedentary lifestyle compared to groups with an active lifestyle. Interestingly, no difference in reserve respiratory capacity was observed between young and old in the active group [97, 102]. Studies on skeletal muscle biopsies from healthy young (age 18–43 years) and older (aged 59–76) have shown that there is an age-related decline in mtDNA content [97, 101, 103]. However, the mtDNA levels of groups leading an active lifestyle were significantly higher compared to the inactive, even though the age-related decline still persisted [97, 101]. Furthermore, the ADP and inorganic phosphate substrate concentrations of the ATP synthase has been shown to be significantly higher in inactive individuals compared to active as well as in elderly compared to young indicating an age-related and sedentary lifestyle associated decrease in ATP synthase activity [95, 102]. Another study of the relative abundance of proteins involved in key metabolic processes in human skeletal muscle revealed a significant age-related decline in 16 proteins involved in the oxidative phosphorylation in the inactive persons. In the active group only subunits of complex IV and an aminotransferase were less abundant in old compared to young [97]. An immediate effect of physical exercise has also been shown. Eight healthy elderly men and women with a sedentary lifestyle were subjected to a 12 week exercise training program. The investigation showed a 53% increase in skeletal muscle mtDNA content after the 12 week program, as well as an almost doubling of ETC activity from complex I through IV and a 62% increase of ETC activity from complex II through IV. The investigations show that a sedentary lifestyle can cause sarcopenia. However, the age-related decline in respiratory capacity can be avoided or reversed by regular endurance exercise [101].

## 9. Conclusion

Generation of ATP by mitochondrial respiration is in many tissues an indispensible source of energy. Many cells and tissues operate at a basal level that only requires a part of their total bioenergetic capability, allowing a reserve respiratory capacity for sudden surges in energy requirement. If the energy requirement supersedes what the reserve respiratory capacity can provide, the cell and the affected tissue risk cell death and organ failure. A decline of the reserve respiratory capacity can increase the incidence of a range of pathologies normally associated with aging. As reviewed, this is evidenced in mouse models having a higher mtDNA mutation rate [15, 16, 93] and in inherited mitochondrial diseases [8]. Mutations of the mtDNA exclusively affect the components of the ETC and the ATP synthase. As described in this paper a disruption of both the control and efficiency of especially complex IV has a significant effect on the short- and long-term regulators of the oxidative phosphorylation, and in extension, the reserve respiratory capacity.

Aging affects most components of the oxidative phosphorylation, which is mediated both by an age-related decline of mtDNA and an age-related decreased expression of nuclear encoded subunits. Of the two genomes, mtDNA has been demonstrated to be most susceptible to age-related accumulation of mutations and deletions [57]. As a result, tissues including brain, heart, and skeletal muscle are subject to an age-related deterioration with a cooccurring loss of both activity and control of the oxidative phosphorylation.

Neuronal tissue is a tissue that is highly dependent of oxidative phosphorylation. This is evidenced by the fact that neurons are demonstrated to use up to 80% of maximal respiratory capacity when firing [67]. In heart tissue, the reserve respiratory capacity plays a pivotal role during acute and chronic stress of the heart. A variety of heart conditions were demonstrated to exhaust the reserve respiratory capacity, leading to cell death when depleted [4, 81–84]. In skeletal muscle, loss of reserve respiratory capacity has been directly related to sarcopenia [93]. With an age-related loss of both activity and control of the oxidative phosphorylation the maximal respiratory capacity will decrease in an age-related manner. This lowers the reserve respiratory capacity of the affected tissues and sensitizes them to sudden surges of ATP demand. As a result the risk of a number of pathologies manifesting in the tissue will be increased.

In the three long-lived postmitotic cell types described in this paper, neurons, heart cells and skeletal muscle cells, cell death, and senescence can be caused by an exhaustion of the reserve respiratory capacity due to mitochondrial dysfunction or aging. This can result in organ failure and have fatal consequences. In contrast to heart and brain, an age-related decline of oxidative phosphorylation has been demonstrated to be reversible in skeletal muscle [101]. An attractive explanation to this could be that skeletal muscles can be regenerated from adult stem cells containing intact mitochondria to a larger extent than what is possible for brain and neurons.
